# Trends in urine screening positive for cannabis of emergency department admissions in Israel 2016–2024

**DOI:** 10.1186/s42238-025-00379-4

**Published:** 2025-12-23

**Authors:** Oren Miron, David Zeltser, Shaul Schreiber, Miriam Adelson, Einat Peles

**Affiliations:** 1 School of Public Health, Health Policy and Management Department, Ben- Gurion University of the Negev, Beer Sheva, Israel; 2 Department of Emergency Medicine, Tel-Aviv Sourasky Medical Center, Tel Aviv, Israel; 3 Gray Faculty of Medical & Health Sciences, Tel Aviv University, Tel Aviv, Israel; 4 Dr. Miriam & Sheldon G. Adelson Clinic for Drug Abuse, Treatment & Research, Division of Psychiatry, Tel-Aviv Sourasky Medical Center, Tel Aviv, Israel; 5 Sagol School of Neuroscience, Tel Aviv University, Tel Aviv, Israel; 6 Adelson Clinic, 10 Dafna St, Tel Aviv, 6492805 Israel

**Keywords:** Cannabis, Emergency department, Risk factors, Toxicology screening, Israel

## Abstract

**Background:**

Although recreational cannabis is not yet legalized in Israel, the increase in cannabis prescriptions may lead to an increase in its availability and abuse.

**Aims:**

To evaluate temporal changes in the prevalence of cannabis-positive urine toxicology tests among individuals admitted to the emergency department (ED) in a large tertiary medical center in Israel.

**Methods:**

Between January/2016 and June/2024, all ED-admitted individuals who had urine toxicology tests (*n* = 20,022) were included.

**Results:**

Screening with urine toxicology increased from 0.7% in 2016 to 1.3% in 2024 (*p* < 0.001). Of all screened patients, 16.8% (*N* = 3361) were cannabis-positive, rising from 15.4% (*n* = 112) in January-June/2016 to 17.6% (*n* = 246) in January-June/2024 (*p* < 0.001), with an increasing trend (*p* = 0.003) among the aged 25-64y group (*n* = 11901) from 16.6% in January-June/2016 to 22.4% in January-June/2024, and the highest proportion in < 25y age group (*n* = 2935, 23.8%). Logistic regression showed that later years (OR = 1.03, 95%CI 1.01–1.05), male sex (OR = 1.5, 95%CI 1.4–1.6), younger age (compared with aged ≥ 65, aged < 25, (OR = 4.1, 95%CI 3. 5-4.8), aged 25–64 (OR = 3.3, 95% CI 2.9–3.8)), Israeli-born (OR = 1.3, 95% CI 1.2–1.4), screening positive for opioid(OR = 1.4, 95%CI 1.2–1.5), MDMA (OR = 1.8, 95%CI 1.5–2.2), cocaine (OR = 1.3, 95%CI 1.2–1.5) methamphetamine (OR = 1.4, 95%CI 1.1–1.7) and not being hospitalized (OR = 1.4, 95%CI 1.3–1.5).

**Conclusions:**

The exponential increase in cannabis prescriptions was not reflected in the modest increase in the proportion of cannabis-positive urine toxicology samples, that characterized the 25–64 age group. However, results may be biased, as toxicology screens were based on clinical suspicion without standardized ordering criteria, and diagnostic information regarding the ED visit was absent.

**Supplementary Information:**

The online version contains supplementary material available at 10.1186/s42238-025-00379-4.

## Introduction

Cannabis is one of the most consumed psychoactive substances worldwide (UNODC, 2023), which is an increasing global health concern (Hammond et al. 2020), partly because over the years, the potency in the world has increased from a mean delta-9-THC concentration of 4% in 1995 to 14% in 2019 (ElSohly et al. 2021). This potency may be even higher, as these estimates are based on samples seized by law enforcement, which is not the entire universe of available cannabis products (excludes legal sources). The increase in potency has led to concerns regarding the development of cannabis use disorder (CUD), which has been reported to range between 8 and 22% among lifetime cannabis users, but may exceed 30% in daily or near daily use (Compton et al. 2019; Kritikos et al. 2025), with increased risk reported for men, individuals with comorbid psychiatric disorders, and those suffering from adverse childhood events (Feingold et al. 2020; Leung et al. 2020). The expansion of recreational cannabis legalization around the world is, has been associated in some studies with increases in hospitalizations, emergency department visits, potential increases in serious/violent crimes, and other harms (Athanassiou et al. 2023). However, varying legalization models may show different impacts on population-level outcomes. A study that evaluated legislation and perceptions in Israel and the USA (Cui et al. 2023) found that lower perceived risk and greater perceived social norms were associated with current use, greater use intentions, and greater intentions to use at home or near children if legalized. In addition, compared with the delta-9-THC, synthetic cannabinoid receptor agonists often have higher potency and efficacy at CB1 and can cause serious adverse effects (Lapoint et al. 2011; Schreiber et al. 2019).

**Fig. 1:**
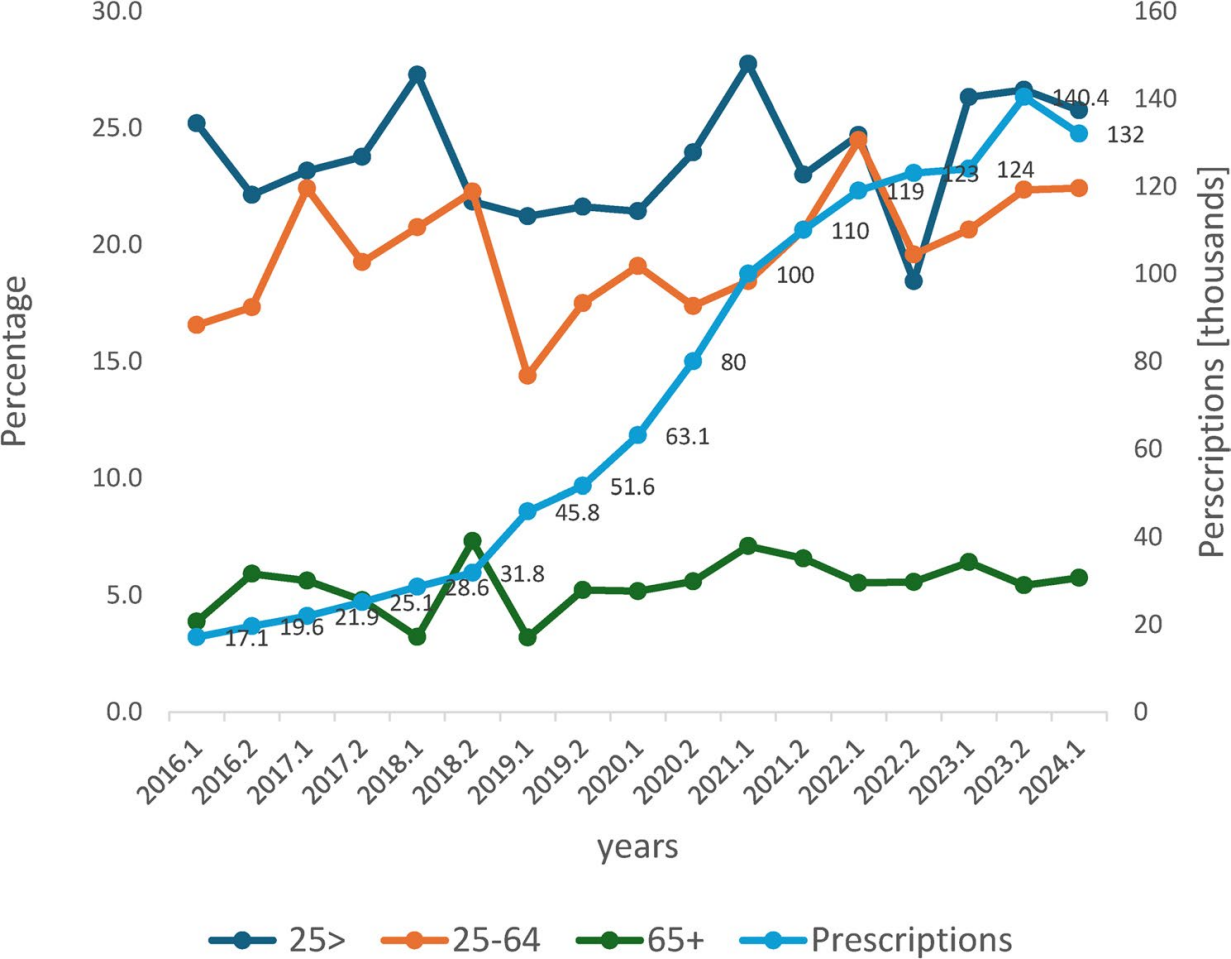
Number of cannabis licenses and percentage of individuals screening positive for cannabis by age group and years (2016-2024).*Legend: *Right y-axis: Number of cannabis licenses by the Ministry of Health. Left y-axis: Percentage of individuals screening positive for cannabis. The 0.1 and 0.2 represent the 1st and 2nd half years. Age 25-64, linear association, p=0.003

In Israel, the Israel Medical Cannabis Agency was established by the Ministry of Health (MOH) in 2011 to regulate medicinal cannabis licensing (Isralowitz et al. 2021). Patient licenses increased substantially from about 600 in 2011 to 16,000 in 2016, 60,000 in 2020, and reached 140,000 in December 2023 (Fig. 1). The indication for more than half of patients was noncancer chronic pain (MOH, (MOH 2025)). Non-medical cannabis use for adults (≥ 18 years old) was decriminalized on April 1, 2019 (Dangerous Drugs Law, 2019) . In 2017, 27% of Israeli adults reported past-year cannabis use (Ezrachi et al. 2017), a substantial increase from the 9% reported in 2009 (Bar-Hamburger et al. 2009).

While recreational use is not yet legalized in Israel, the substantial increase in cannabis prescriptions may reflect a ‘back-door legalization’, which may be reflected among ED-admitted individuals. We aimed to study the proportion of cannabis use between 2016 and 2024 among individuals admitted to the largest (1500 beds) teaching academic Hospital ED in Tel Aviv, serving a catchment area of more than a million people, and to characterize individuals who used cannabis only versus those who used cannabis plus other substances. We hypothesized that the increase in licensed medical cannabis use would be paralleled by a rise in cannabis-positive ED toxicology screens, particularly among younger patients, and individuals with cannabis only use would show better outcomes. We studied among clinically suspected individuals who underwent toxicology screening, at-risk group; the presence of other substances, information on blood-transmitted disease, and prescribed medications for anxiety and depression, which are prevalent among individuals with substance use disorder (Choi et al. 2021).

## Methods

The Helsinki Committee standard (IRB) of Tel Aviv Sourasky Medical Center (TASMC) is in accordance with the Declaration of Helsinki. The TASMC Helsinki Committee approved the study analyses (approval number TASMC-0503-22). Human Ethics and Consent to Participate declarations are not applicable, as only de-identified retrospective data were included.

### Data source

De-identified admissions data were retrieved from the TASMC emergency department (ED) database using an electronic format.

### Patient selection

All individuals admitted to the ED between January 1, 2016, and June 30, 2024, who were screened with urine toxicology at ED admissions (had code UTOX) were included in the data analyses. The decision to request a urine toxicology test is a clinical one, with no pre-set criteria for ordering it. The ED diagnosis was available since 2017 and contained mostly ICD codes or signs and symptoms (see distribution in the Appendix).

### Toxicology test procedure

The multi-drug 2–17 Drugs Rapid Test Cassette (Urine) (Acro Biotech, USA) is a lateral flow immunoassay designed for the qualitative detection of multiple drugs and their metabolites in human urine. The test utilizes monoclonal antibodies to selectively detect elevated levels of specific drugs. Drugs included: Methamphetamine (calibrator d-Methamphetamine, cut-off 1000ng/ml); Cocaine (calibrator Benzoylecgonine, cut-off 300ng/ml); Marijuana/Cannabinoid (calibrator 11 nor THC 9 COOH, cut-off 50 ng/ml); MDMA (ecstasy) (calibrator d,1 MDMA, cut-off 500 ng/ml); opioids including methadone (calibrator methadone, cut-off 300 ng/ml); opiates (calibrator morphine, cut-off 300 ng/ml) and Oxycodone (calibrator oxycodone, cut-off 100 ng/ml); Benzodiazepines (calibrator Oxazepam, cut-off 300 ng/ml); Tricyclic antidepressants (calibrator nortriptyline, cut-off 1000 ng/ml); Barbiturates (calibrator Secobarbital, cut-off 300 ng/ml); PCP (Phencyclidine) (calibrator Phencyclidine, cut-off 20 ng/ml); and Amphetamines (calibrator d-Amphetamine, cut-off 1000 ng/ml). Fentanyl was qualitatively detected using the ARK™ Fentanyl II Assay (ARK Diagnostics, Inc.), a homogeneous enzyme immunoassay (EIA) with a 1.0 ng/mL cutoff, executed on Siemens ADVIA^®^ 2400 Chemistry Systems.

Diagnosis information included ICD codes or signs and symptoms (available since 2017). Sociodemographic variables included: sex, age, Israeli born, past diagnosis of blood-transmitted disease that are known to characterize individuals with substance use disorder (antibodies for HIV and hepatitis C, antigen for hepatitis B), and prescription medication use (opioids, benzodiazepines that may be abused, and antidepressants (SSRI). Hospitalization (not discharge from ED), and death within 7 days of ED admission were also included in the analyses. Vital status was determined from the Israel National Population Registry, which records all deaths in the country and can be accessed from TASMC.

### Statistical analyses

Data analysis was performed using SPSS version 29. We calculated the proportion of emergency department admissions that underwent a drug test. Significant trend changes by half-year intervals were tested using Pearson chi-square or linear-by-linear association of all screened subjects and by age group (< 25, 25–64, ≥ 65) separately. In univariate analysis, significant differences were compared using the chi-square or Fisher Exact tests for categorical variables, and t-test or analyses of variance (ANOVA) for continuous variables. Comparisons included individuals screening positive vs. screening negative for cannabis (Table 1), screening positive for cannabis only vs. for cannabis plus other substance (Table 2) and also compared to screening negative for cannabis (Table 2), and comparison between 5 groups: screening positive for cannabis only, cannabis plus other substance, negative for cannabis and positive for other one substance, negative for cannabis and positive for more than one other substances, and negative for all substances (Table 3). Multivariate logistic regression (Forward Conditional Stepwise Analyses) was used to compare group models (cannabis tested positive vs. negative; cannabis only vs. cannabis plus other substance; non-cannabis other one substance vs. non-cannabis other polysubstance), including variables that were significant in univariate analyses (*p* < 0.1). The variables are listed in the results, multivariate analyses section. The Hosmer-Lemeshow test was used to assess logistic regression model goodness-of-fit, with *p* > 0.05 indicating adequate fit.

**Table 1:** Characteristics of individuals screening positive vs. negative for cannabis (2016–2024)

	Cannabis 3361(100%)	No cannabis 16,661(100%)	*P* value*
Male, sex	2229(66.3)	9300(55.8)	**< 0.001**
Age, y	38.2 ± 15.8	50.1 ± 21.7	**< 0.001**
Age groups, y < 25 25–64.9 ≥ 65	698(20.8) 2372(70.7) 286(8.5)	2237(13.5) 9529(57.4) 4832(29.1)	**< 0.001**
Israeli born	2266 (67.4%)	9303 (55.8)	**< 0.001**
Hepatitis C antibody	653(19.4)	3562(21.4)	**0.012**
HIV antibody	7(0.2)	33(0.2)	0.8
Hepatitis B antigen	298(8.9)	1464(8.8)	0.9
Prescribed medications			
Opioid	141(4.2)	589(3.5)	0.07
Benzodiazepine	267(7.9)	1380(8.3)	0.5
SSRI	144(4.3)	975(5.9)	**< 0.001**
Urine Toxicology			
Any opioids	476(14.2)	1824(10.9)	**< 0.001**
MDMA (Ecstasy)	209(6.2)	362(2.2)	**< 0.001**
Cocaine	386(11.5)	963(5.8)	**< 0.001**
Benzodiazepines	741(22.0)	3649(21.9)	0.9
Amphetamines	146(4.3)	361(2.2)	**< 0.001**
Methamphetamines	198(5.9)	413(2.5)	**< 0.001**
Phencyclidine	23(0.7)	75(0.5)	0.07
Tricyclic antidepressant	109(3.3)	491(3.0)	0.3
Barbiturate	36(1.1)	191(1.1)	0.8
Treatment & Outcome			
Hospitalized	1793(53.3)	11,333(68.0)	**< 0.001**
Died (within 7 days)	25(0.7)	315(1.9)	**< 0.001**

*Categorical variables compared using Chi-square or Fisher’s exact test. Continuous variable (age) compared using an independent samples t-test. Age groups compared using the Chi-square test

**Table 2:** Characteristics of individuals screening positive for cannabis only, cannabis & other substances, and screening negative for cannabis

	Cannabis			No cannabis	
	Cannabis only	Cannabis & others	*P* value*	Cannabis negative	*P* value*#
	1871(100%)	1490(100%)		16,661(100%)	
Male, sex	1238(66.2)	991(66.5)	0.9	9300(55.8)	**< 0.001**
Age, y	37.3 ± 15.4	39.5 ± 16.8	**< 0.001**	50.1 ± 21.7	**< 0.001**
Age group < 25 25–64.9 ≥ 65	435(23.3) 1295(69.3) 139(7.4)	263(17.7) 1077(72.4) 147(9.9)	**< 0.001**	2237(13.5) 9529(57.4) 4832(29.1)	**< 0.001**
Israeli born	1284 (68.6%)	982(65.9)	0.1	9303 (55.8)	**< 0.001**
Hepatitis C antibody	294(15.7)	359(24.1)	**< 0.001**	3562(21.4)	**< 0.001**
HIV antibody	2(0.1)	5(0.3)	0.3	33(0.2)	0.3
Hepatitis B antigen	140(7.5)	158(10.6)	**0.002**	1464(8.8)	**0.007**
Prescribed medications					
Opioid	34(1.8)	107(7.2)	**< 0.001**	589(3.5)	**< 0.001**
Benzodiazepine	86(4.6)	181(12.1)	**< 0.001**	1380(8.3)	**< 0.001**
SSRI	64(3.4)	80(5.4)	**0.006**	975(5.9)	**< 0.001**
Urine Toxicology					
Any opioids	-	476(31.9)		1824(10.9)	**< 0.001**
MDMA (Ecstasy)	-	209(14.0)		362(2.2)	**< 0.001**
Cocaine	-	386(25.9)		963(5.8)	**< 0.001**
Benzodiazepines	-	741(49.7)		3649(21.9)	**< 0.001**
Amphetamines	-	146(9.8)		361(2.2)	**< 0.001**
Methamphetamines	-	198(13.3)		413(2.5)	**< 0.001**
Phencyclidine	-	23(1.5)		75(0.5)	**< 0.001**
Tricyclic	-	109(7.3)		491(3.0)	**< 0.001**
Barbiturate	-	36(2.4)		191(1.1)	**< 0.001**
Treatment & Outcome					
Hospitalized	955(51.0)	838(56.2)	**0.003**	11,333(68.0)	**< 0.001**
Died (within 7 days)	16(0.9)	9(0.6)	0.4	315(1.9)	**< 0.001**

*Categorical variables are compared using the Chi-square or Fisher’s exact test. Continuous variable (age) compared using t-test for two-group comparisons (cannabis only vs. cannabis & others) and ANOVA for the three groups. #Comparison between the three groups

**Table 3:** Characteristics of 5 subgroups: cannabis only, cannabis & other substances, one other substance, poly other substances, and negative to any substance

	Cannabis		No Cannabis			
	Cannabis only	Cannabis & other substances	One other substance	Poly other substances	Negative to any substance	*P* value*
	1871(100%)	1490(100%)	4293(100%)	1738(100%)	10,630(100%)	
Male, sex	1238(66.2)	991(66.5)	2288(53.3)*	1027(59.1)	5985(56.3)	**< 0.001**
Age, y	37.3 ± 15.4*	39.5 ± 16.8	52.3 ± 21.2*	49.6 ± 19.0	49.2 ± 22.2	**< 0.001**
Age group < 25 25–64.9 ≥ 65	435(23.3)* 1295(69.3) 139(7.4)	263(17.7) 1077(72.4) 147(9.9)	403(9.4)* 2537(59.2) 1344(31.4)	135(7.8) 1194(69.2) 396(23.0)	1699(16.0) 5798(54.8) 3092(29.2)	**< 0.001**
Israeli born	1284 (68.6)	982(65.9)	2370(55.2)#	1008(58.0)	5925 (55.7)	**< 0.001**
Hepatitis C Ab	294(15.7)*	359(24.1)	1009(23.5)*	536(30.8)	2017(19.0)	**< 0.001**
HIV antibody	2(0.1)	5(0.3)	9(0.2)	9(0.5)	15(0.1)	**0.036**
Hepatitis B Ag	140(7.5)	158(10.6)	416(9.7)*	229(13.2)	819(7.7)	**< 0.001**
Prescribed medications						
Opioid	34(1.8)*	107(7.2)	230(5.4)*	186(10.7)	173(1.6)	**< 0.001**
Benzodiazepines	86(4.6)*	181(12.1)	527(12.3)*	281(16.2)	572(5.4)	**< 0.001**
SSRI	64(3.4)*	80(5.4)	327(7.5)*	88(5.1)	563(5.3)	**< 0.001**
Urine Toxicology						
Any opioids	-	476(31.9)	824(19.2)*	1000(57.5)	-	
MDMA	-	209(14.0)	139(3.2)*	223(12.8)	-	
Cocaine	-	386(25.9)	344(8.0)*	619(35.6)	-	
Benzodiazepines	-	741(49.7)	2492(58.1)*	1157(66.6)	-	
Amphetamines	-	146(9.8)	111(2.6)*	250(14.4)	-	
Meth-amphetamine	-	198(13.3)	84(2.0)*	329(18.9)	-	
Phencyclidine	-	23(1.5)	28(0.7)*	47(2.7)	-	
Tricyclic	-	109(7.3)	208(4.9)*	283(16.3)	-	
Barbiturate	-	36(2.4)	63(1.5)*	128(7.4)	-	
Treatment & Outcome						
Hospitalized	955(51.0)*	838(56.2)	2954(68.8)*	1127(64.8)	7252(68.2)	**< 0.001**
Died (≤ 7 days)	16(0.9)	9(0.6)	86(2.0)*	38(2.2)	191(1.8)	**< 0.001**

P values in the column compare all 5 groups using Chi-square (categorical) or ANOVA (continuous). Asterisks (*) indicate p < 0.01 for specific pairwise comparisons: Column ‘Cannabis only’ compares cannabis only vs. cannabis & others; Column ‘One other substance’ compares one vs. poly other substances (p values using Fishers exact test (categorical) or t-test (continuous). # indicates *p* < 0.05

## Results

### Positive cannabis screening test, prevalence and trends

The study included 20,022 individuals admitted to the ED between 2016 and June 2024 who were screened for toxicology drugs. During that period, the overall number of ED admissions did not change and was about 100,000 per year. However, the proportion of admitted individuals who underwent toxicological testing nearly doubled from 0.7% in January-June 2016 to 1.3% in January-June 2024 (linear association, *p* < 0.001). Of all screened patients, 3361 (16.8%) tested positive for cannabis. Their proportion increased from 15.4% in January-June 2016 to 17.6% in January-June 2024 (Pearson chi-square, *p* < 0.001). We stratified cannabis proportion by 3 age groups: <25, 25–64, and ≥ 65 years. These age groups differed significantly by proportion of hospitalization (41.8%, 61.5%, 88.7% respectively, linear association *p* < 0.001) and mortality within 7 days (0.3%, 1.2%, 4.5% respectively, linear association *p* < 0.001).

The cannabis proportion was highest (23.8%) in younger patients (< 25 y) compared with the 25–64 age group (19.9%) and ≥ 65 age group (5.6%, chi-square *p* < 0.001). However, a significant increasing trend was observed among the 25–64 y group only, from 16.6% in 2016 to 22.4% in 2024 (linear association 9.0, *p* = 0.003), with no significant linear association trend for aged group < 25 (*p* = 0.5) or ≥ 65 (*p* = 0.3) (Fig. 1).

### Positive of other substance screening trends

**Fig. 2:**
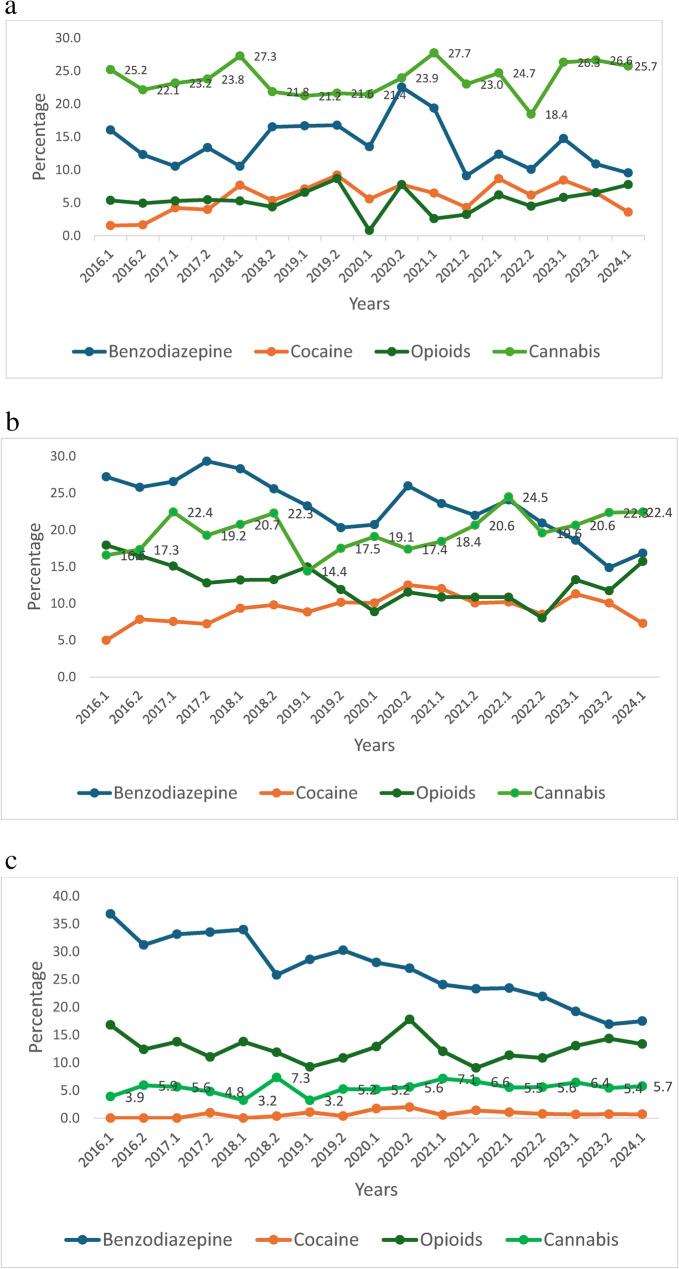
**a **Percentage of individuals screening positive for drugs by years (Age <25 y).*Legend: *The 0.1 and 0.2 represent the 1st and 2nd half years. Cocaine - linear association p=0.048. **b** Percentage of individuals screening positive for drugs by years (Age 25-64 y).*Legend: *The 0.1 and 0.2 represent the 1st and 2nd half years. Benzodiazepine - linear association p<0.001; Cocaine - linear association p=0.011; Cannabis - linear association, p=0.003. **c** Percentage of individuals screening positive for drugs by years (Age ≥65 y).*Legend: *The 0.1 and 0.2 represent the 1st and 2nd half years. Benzodiazepine - linear association p<0.001

The percentage of individuals testing positive for benzodiazepines decreased from 27.2% in January-June 2016 to 16.2% in January-June 2024 (linear association *p* < 0.001), while the percentage of individuals testing positive for cocaine increased from 3.3% to 4.8%, with a peak of 8.9% in July-December 2020 (linear association *p* = 0.052). Positive proportion for opioids decreased from 15.4% in January-June 2016 to 8.3% in January-June 2022 (linear association, *p* < 0.001), but significantly increased from 8.3% in June-December 2022 to 14% in January-June 2024 (linear association *p* < 0.001). The fentanyl test was added to the toxicology panel in January 2023. Stratified by age groups, the 25–64 and ≥ 65 age groups (Fig. 2b, c) showed benzodiazepine positive screening tests reduced (linear association *p* < 0.001 for each), cocaine positive screening tests increased in age groups < 25y and 25-64y (linear association *p* = 0.048 and *p* = 0.011 respectively), and opioids positive screening tests decreased in the 25-64y age group (linear association *p* = 0.001) (Fig. 2a-c).

### Characteristics of being positive for cannabis (Table 1)

Compared with the screened negative group (*n* = 16,661), those who screened positive for cannabis (*n* = 3361, 16.8%) were younger (38.2 ± 15.8 vs. 50.1 ± 21.7, *p* < 0.001), more of them were male (*p* < 0.001), Israeli born (*p* < 0.001), fewer had hepatitis C antibody (*p* = 0.012) and had SSRI prescriptions (*p* < 0.001). More of them tested positive for any opioids (*p* < 0.001), cocaine (*p* < 0.001), MDMA (ecstasy) (*p* < 0.001), amphetamines (*p* < 0.001), and methamphetamines (*p* < 0.001), and fewer of them were hospitalized (*p* < 0.001) and died within 7 days since ED admission (*p* < 0.001).

Among the 2935 young patients aged < 25y, compared with the screened negative group (*n* = 2237), those screening positive for cannabis (*n* = 698, 23.8%) had higher proportion of male (58% vs. 45%, *p* < 0.001), lower proportion of Israeli born (74.8% vs. 79.7%, *p* = 0.007), more of them tested positive for any opioids (8.5% vs. 4.5%, *p* < 0.001), cocaine (10.7% vs. 4.4%, *p* < 0.001), MDMA (ecstasy) (6.7% vs. 3.6%, *p* < 0.001), amphetamines (5.0% vs. 2.4%, *p* < 0.001) methamphetamines (7.6% vs. 2.9%, *p* < 0.001), benzodiazepine (18.8% vs. 12.1%, *p* < 0.001) and had opioid prescription (1.6% vs. 0.5%, *p* = 0.009). Hospitalization and death within 7 days were comparable (*p* = 0.2 and *p* = 0.7, respectively).

Similarly, among the 11,901 aged 25–64 y group, compared with the screened negative group (*n* = 9529), those screening positive for cannabis (*n* = 2372, 19.9%) were more likely to be male (70.1% vs. 61.4%, *p* < 0.001), Israeli born (67.6% vs. 59.3%, *p* < 0.001), and tested positive for opioids (14.5% vs. 12.0%, *p* = 0.001), cocaine (12.9% vs. 8.6%, *p* < 0.001), MDMA (ecstasy) (6.5% vs. 2.6%, *p* < 0.001), amphetamines (4.5% vs. 2.3%, *p* < 0.001) methamphetamines (5.8% vs. 2.9%, *p* < 0.001), but not to benzodiazepine (22.0% vs. 22.8%, *p* = 0.4) or opioid prescription (3.4% vs. 3.2%, *p* = 0.6). Unlike the < 25 age group, a lower proportion of hepatitis C seropositive (21% vs. 24%, *p* = 0.002), hospitalized individuals (54.3% vs. 63.5%, *p* < 0.001), and death within 7 days (0.6% vs. 1.2%, *p* = 0.018) were observed among patients screening positive for cannabis.

The ≥ 65 aged group included 5118 individuals. Compared with the screened negative group (*n* = 4832), those screening positive for cannabis (*n* = 286, 5.6%) were comparable by male proportion (55.2% vs. 49.6%, *p* = 0.068), and more were Israeli-born (49% vs. 38.5%, *p* < 0.001). Except for cocaine (1.4% vs. 0.7%, *p* = 0.3), amphetamines (1.4% vs. 1.7%, *p* = 1), and methamphetamines (2.1% vs. 1.4%, *p* = 0.3), more of them tested positive for opioids (25.2% vs. 11.7%, *p* < 0.001), MDMA (ecstasy) (3.1% vs. 0.6%, *p* < 0.001), benzodiazepine (30.1% vs. 24.7%, *p* = 0.049), and tricyclic antidepressants (5.6% vs. 3.1%, *p* = 0.037). More had benzodiazepine prescriptions (13.3% vs. 8.4%, *p* = 0.007), opioids (17.1% vs. 5.7%, *p* < 0.001), and SSRI (15.7% vs. 10.8%, *p* = 0.012). Hepatitis C seropositivity (22.7% vs. 21.5%, *p* = 0.6) and death within 7 days (2.8% vs. 4.0%, *p* = 0.4) did not differ between groups, but fewer were hospitalized (79.7% vs. 89.2%, *p* < 0.001).

### Cannabis only vs. cannabis and other substance users (Table 2)

We also compared individuals who tested positive only for cannabis (*n* = 1871, 55.7%) to those who tested positive for cannabis and other substances (*n* = 1490, 44.3%). Individuals who were only positive for cannabis were younger (*p* < 0.001), fewer of them had hepatitis C antibodies (*p* < 0.001), and hepatitis B antigen (*p* = 0.002). Also, fewer of those who tested positive for cannabis and other substances had prescriptions for opioids (*p* < 0.001), benzodiazepines (*p* < 0.001), and SSRIs (*p* = 0.006), and were hospitalized (*p* = 0.003). The groups were comparable in sex, Israeli-born, HIV, and death rate.

**Fig. 3:**
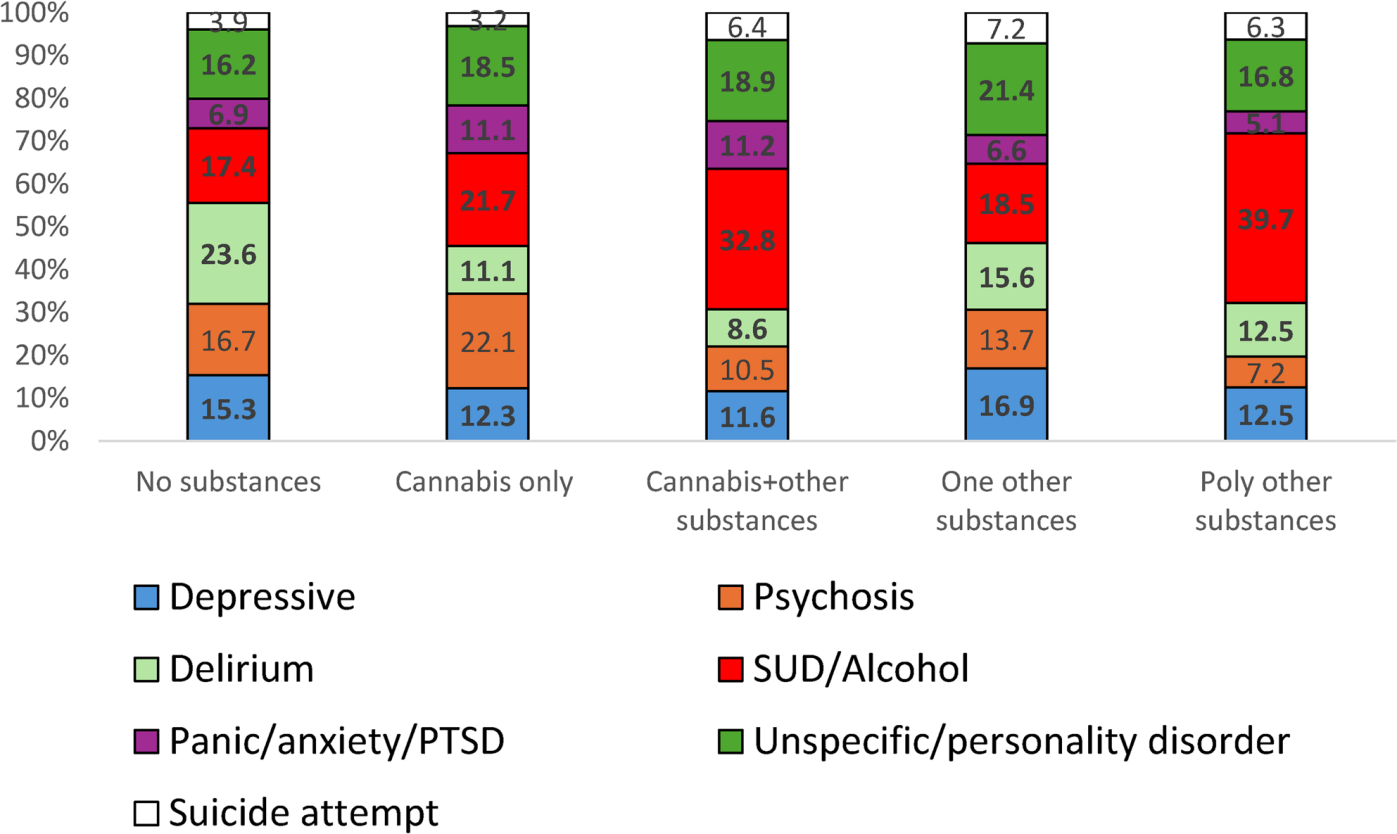
Mental health proportion distribution by substance groups.*Legend: *Groups: no substances, cannabis only, cannabis & other substances, one other substance, and poly other substances. Chi-square p<0.001

Of the non-cannabis group, 6031 individuals tested positive to other substances (4293 tested positive to one other substance and 1738 tested positive to other poly-substances), leaving 10,630 individuals negative to all toxicology-screened tests. The characteristics and comparison between the 5 subgroups are presented in Table 3. The distribution of mental diagnosis subgroups differed significantly among the 5 subgroups (*p* < 0.001) (Fig. 3). The psychosis proportion was highest in the cannabis only group (22.1%), while substance and alcohol use was highest in the two poly-substance groups: 32.8% with cannabis and 39.7% without cannabis.

### Multivariate analyses

A forward conditional stepwise logistic regression model comparing individuals screening negative vs. screening positive for cannabis found later years (OR = 1.03, 95% CI 1.01–1.05, *p* < 0.001), male sex (OR = 1.5, 95% CI 1.4–1.6, *p* < 0.001), younger age (compared with those aged ≥ 65: those aged < 25 OR = 4.1, 95% CI 3.5–4.8, *p* < 0.001; those aged 25–64 OR = 3.3, 95% CI 2.9–3.8, *p* < 0.001), Israeli born (OR = 1.3, 95% CI 1.2–1.4, *p* < 0.001), tested positive for opioid (OR = 1.4, 95% CI 1.2–1.5, *p* < 0.001), for MDMA (ecstasy) (OR = 1.8, 95% CI 1.5–2.2, *p* < 0.001), for cocaine (OR = 1.3, 95% CI 1.2–1.5, *p* < 0.001), for methamphetamine (OR = 1.4, 95% CI 1.1–1.7, *p* = 0.003), and not being hospitalized (OR = 1.4, 95% CI 1.3–1.5, *p* < 0.001) to characterize individuals screening positive for cannabis. The Hosmer-Lemeshow goodness-of-fit test indicated adequate model fit (*p* = 0.1). The model included half a year of screening, sex, age group (< 25, 25–64, ≥ 65), Israeli born, hepatitis C seropositive, having an SSRI prescription, toxicology screening result for opioids, MDMA (ecstasy), cocaine, amphetamines, and methamphetamines, hospitalization, and 7-day mortality.

A forward conditional stepwise logistic regression model comparing individuals screening positive for cannabis only vs. those screening positive for cannabis and other substances, found opioid prescription (OR = 3.2, 95% CI 2.1–4.8, *p* < 0.001), benzodiazepine prescription (OR = 2.4, 95% CI 1.8–3.1, *p* < 0.001), and hepatitis C antibody (OR = 1.6, 95% CI 1.3–1.9, *p* < 0.001) to characterize individuals screening positive for cannabis and other substances. The Hosmer-Lemeshow goodness-of-fit test indicated adequate model fit (*p* = 0.8). The model included half a year of screening, age, hepatitis C, hepatitis B, opioid prescription, benzodiazepine prescription, SSRI prescription, and hospitalization.

A forward conditional stepwise logistic regression model comparing among the individuals screening negative for cannabis, those screening positive for one substance vs. those screening positive for poly-substances, found male sex (OR = 0.9, 95% CI 0.8–1.8, *p* = 0.044), no being hospitalized (OR = 0.8, 95% CI 0.7–0.9, *p* = 0.001), opioid prescription (OR = 2.1, 95% CI 1.7–2.6, *p* < 0.001), benzodiazepine prescription (OR = 1.3, 95% CI 1.1–1.6, *p* = 0.001), not SSRI prescription (OR = 0.7, 95% CI 0.5–0.9, *p* = 0.006), hepatitis C antibody (OR = 1.4, 95% CI 1.2–1.5, *p* < 0.001), and aged 25–64 years group (OR = 1.3, 95% CI 1.0–1.6.0.6, *p* = 0.017) to characterized individuals screening positive for poly-substances. The Hosmer-Lemeshow goodness-of-fit test indicated adequate model fit (*p* = 0.4). The model included age groups, hepatitis C, hepatitis B, opioid prescription, benzodiazepine prescription, SSRI prescription, hospitalization, sex, and Israeli-born.

### Summary of main findings

Over eight and a half years, there was a significant trend toward increased positivity for cannabis over time, which was significant for the 25–64-year age group (from 16.6% to 22.4%, linear association *p* = 0.003). The cannabis proportion in that age group became higher than that of other substances (benzodiazepines, cocaine, and opioids) in the late years between June 2023 and June 2024. Among the < 25 age group, cannabis use was consistently highest throughout all 8.5 years (23.8%). Among the ≥ 65 age group, cannabis proportion was much lower than that of benzodiazepine and opioids (5.6%), with no significant increase from 3.9% in January-June 2016 to 5.7% in January-June 2024. The proportion of toxicology testing also significantly increased over the years; however, each substance showed a different pattern. Unlike the increasing cannabis trend, benzodiazepine showed a trend to decrease; therefore, we cannot attribute the increasing trend of cannabis screening positive tests to the increasing trend of toxicology testing.

In multivariate analyses, individuals screened positive to cannabis were more likely to be male (OR = 1.5), Israeli-born (OR = 1.3), to test positive for opioid (OR = 1.4), cocaine (OR = 1.3), MDMA (ecstasy) (OR = 1.8), and methamphetamine (OR = 1.4), and less likely to be hospitalized (OR = 1.4). Those who screened positive for cannabis only, compared with those who also screened positive for other drugs, were less likely to be hepatitis C seropositive (OR = 1.6) and less likely to have an opioid (OR = 3.2) and a benzodiazepine (OR = 2.4) prescription.

## Discussion

Notably, despite the more than sixfold increase in cannabis prescriptions during the study period (from approximately 16,000 in 2016 to over 140,000 in late 2023), the increase in cannabis-positive toxicology screens was only modest. The time course of medical licensing did not track closely with toxicology results; the exponential growth in licensing was not mirrored by an exponential increase in ED cannabis positivity. This suggests that licensed medical cannabis users are not presenting to the ED at elevated rates, or that the toxicology screening captures a population distinct from those with medical licenses.

Surprisingly, two outcomes that we studied, hospitalization and mortality, were both lower among patients who screened positive for cannabis; mortality within 7 days was lower, although only in univariate analyses. Both the cannabis-only and cannabis polydrug-screened groups had lower mortality within 7 days than individuals who tested positive for other substances but negative for cannabis. As the latter group was older and may have suffered from more severe illness, we compared mortality by age groups and found a non-significant trend toward lower mortality among patients who screened positive for cannabis. Specifically, the 25–64 age group showed significantly lower mortality among cannabis users, while the < 25 and ≥ 65 groups did not significantly differ.

Looking at the literature, a study from Oregon showed that only 1.8% of visits to the ED were cannabis-related, despite concern that cannabis-related visits may pose a larger resource burden to the healthcare system (Hendrickson et al. 2021). A case-control cohort study from Ontario, Canada (2014–2017) found that cannabis users had significantly higher odds of an all-cause ED visit (OR 1.22, 95% CI 1.13–1.31), but the odds of mortality were not affected (Vozoris et al. 2022). Concerning lower hospitalization rates, one study that also found lower hospitalization rates in cannabis users reported a significant increase in individuals leaving the ED against medical advice, which could confound this result (Yeung et al. 2020). We do not have this information.

In our analyses, the comparison between cannabis-only and cannabis-polydrug groups suggests that the cannabis-only group may reflect non-injected drug users with less severe medical presentations. Specifically, the cannabis-only group had significantly lower rates of hepatitis C antibody positivity (15.7% vs. 24.1%, *p* < 0.01), a marker associated with injection drug use. They also had lower rates of prescribed opioids (1.8% vs. 7.2%) and benzodiazepines (4.6% vs. 12.1%), and lower hospitalization rates (51.0% vs. 56.2%). Cannabis testing positive does not necessarily reflect cannabis abuse or cannabis use disorder, and for those who self-reported licensed prescriptions and presented with normal behavior, a toxicology test may not have been requested.

It is important to mention that cannabis use outcomes cover several aspects not reflected in medical outcomes. Recently, a meta-analysis (Chan et al. 2024) reported associations with school absenteeism and dropout, reduced likelihood of obtaining high academic grades, and potentially increased unemployment. A review of mental health adverse events related to cannabis use, as diagnosed in the ED (Crocker et al. 2023) reported concerns regarding the ability to disentangle cannabis use adverse events from adverse events associated with multiple recreational substances.

The strength of our study lies in observations based on big data from the ED of a large tertiary medical center in the largest municipal city in Israel over a long period (eight and a half years), including several toxicology substances that were checked, allowed us to measure cannabis trends while Israel was experiencing the highest increase in cannabis prescriptions in the world during that period.

### Limitations

The major limitations are the absence of set criteria for ordering a toxicology screen, which may lead to sampling bias, and the fact that testing criteria may have changed over time and among clinicians in unknown ways. Another limitation is the absence of diagnostic information regarding the ED visit, including whether it was even cannabis related. Importantly, a positive cannabis toxicology screen does not necessarily indicate that the ED visit was cannabis-related; Shelton et al. (2020) found that only approximately 26% of ED visits with cannabis-positive ICD codes were actually attributable to cannabis. The study lacks data on cannabis dosage and type of cannabis. A comparison of characteristics of patients who were screened vs. those who were not screened was not available, which is an additional limitation.

## Conclusions

An elevation in cannabis-positive urine toxicology samples among the 25–64 age group may reflect the increase in cannabis use in the general population. However, during that period, while the number of cannabis prescriptions increased by more than sixfold, only a significant yet modest increase in cannabis positivity was observed. The outcomes of those who screened positive were better (fewer hospitalizations and deaths). However, additional outcomes, as well as later outcomes (i.e., cannabis use disorder, psychosis, pain, and mental health comorbidity), would provide important insights regarding cannabis use.

## Supplementary Information

Supplementary Material 1.

## Data Availability

Data cannot be shared openly but is available from the authors upon reasonable request.
