# Supplementary material for: Trends in urine screening positive for cannabis of emergency department admissions in Israel 2016–2024
Source: J Cannabis Res. 2025 Dec 23;8:14. doi: 10.1186/s42238-025-00379-4 (PMC12838475; doi:10.1186/s42238-025-00379-4)
Supplement: Supplementary file 1 — Supplementary Material 1. [file 42238_2025_379_MOESM1_ESM.docx]

Appendix

| perinatal period condition | 4 |
| --- | --- |
| rape etc. | 5 |
| poisoning drug 850-858 | 7 |
| 860-879 poisoning other | 7 |
| Congenital abnormality | 12 |
| pregnancy, childbirth problem | 24 |
| blood and blood forming organ | 57 |
| Neoplasm | 73 |
| 960-999 homicide and others | 85 |
| Donors of other specified organ or tissue | 102 |
| surgical and other procedure | 140 |
| observation after accident | 146 |
| skin and subcutaneous | 155 |
| infectious & parasites | 159 |
| musculoskeletal system | 190 |
| Motor transport accident 0-849 | 193 |
| 950-959 suicide | 276 |
| Digestive system | 278 |
| falls 880-888 | 320 |
| Genitourinary System | 428 |
| Endocrine, metabolic | 481 |
| Respiratory system | 498 |
| Observation for unspecified suspected condition | 544 |
| circulatory system | 1053 |
| injury and poisoning | 1261 |
| nervous system | 1609 |
| symptoms, signs, and defined condition | 4939 |
| Mental disorder | 5157 |

Appendix figure

Distribution of mental health proportion diagnosis by half years (2017-2024)
